# Geriatric nutritional risk index as a predictor of complications and long-term outcomes in patients with gastrointestinal malignancy: a systematic review and meta-analysis

**DOI:** 10.1186/s12935-020-01628-7

**Published:** 2020-10-31

**Authors:** Hailun Xie, Shuangyi Tang, Lishuang Wei, Jialiang Gan

**Affiliations:** 1 Department of Colorectal and Anal Surgery, The First Affiliated Hospital, Guangxi Medical University, 6 Shuangyong Road, Nanning, Guangxi 530021 China; 2 Department of Pharmacy, The First Affiliated Hospital, Guangxi Medical University, Nanning, Guangxi P.R. China; 3 Geriatric Respiratory Disease Ward, The First Affiliated Hospital, Guangxi Medical University, Nanning, Guangxi P.R. China

**Keywords:** Geriatric nutritional risk index, Complications, Prognosis, Gastrointestinal malignancy

## Abstract

**Background:**

The effect of the geriatric nutritional risk index (GNRI) on the prognosis of patients with gastrointestinal malignancy remains unclear. The aim of our study was to systematically explore the value of the GNRI in evaluating postoperative complications and long-term outcomes in gastrointestinal malignancy.

**Methods:**

A systematic literature search was conducted using electronic databases to report the impact of the GNRI on postoperative complications and long-term outcomes of patients with gastrointestinal malignancies as of August 2020. The hazard ratio (HR) with a 95% confidence interval (CI) was used to evaluate the impact of the GNRI on long-term outcomes. The risk ratio (RR) with 95% CI was used to assess the impact of the GNRI on postoperative complications.

**Result:**

A total of nine studies with 2,153 patients were enrolled in our meta-analysis. The results suggested that a low GNRI was correlated with poor overall survival of patients with gastrointestinal malignancy (HR = 1.94, 95% CI 1.65–2.28, p < 0.001). Patients with a low GNRI had a higher risk of complications than patients with a high GNRI (OR = 2.19, 95% CI 1.57–3.05, p < 0.001). In addition, patients with a low GNRI had shorter relapse-free survival (HR = 2.45, 95% CI 1.50–4.00, p < 0.001) and disease-free survival (HR = 1.84, 95% CI 1.23–2.76, p = 0.003) than those with a high GNRI. However, the GNRI was not an independent factor affecting cancer-specific survival (HR = 1.60, 95% CI 0.91–2.82, p = 0.101).

**Conclusion:**

Based on existing evidence, the GNRI was a valuable predictor of complications and long-term outcomes in patients with gastrointestinal malignancy.

## Background

Gastrointestinal (GI) malignancy is a form of malignancy that occurs in the gastrointestinal tract and its accessory organs, accounting for nearly 30% of cancer incidence and 32% of cancer deaths worldwide [1, 2]. Although significant progress has been made in the individualized treatment of cancer patients, the clinical efficacy of most patients with GI malignancy is still poor. A number of biomarkers have been reported as prognostic factors for GI malignancy. However, these biomarkers are concentrated mainly in histochemistry and molecular biology techniques. Their application is limited to a certain extent because of the high price and need for specific experimental equipment [3, 4]. Therefore, it continues to be important to find convenient, effective, and inexpensive prognostic markers for patients with GI malignancy.

Cancer patients, especially those with GI malignancy, are prone to malnutrition. The prevalence of malnutrition in cancer patients ranges from 20 to 70%, while in GI malignancy patients, the prevalence is as high as 80% [5–7]. Many studies have shown that malnutrition in cancer patients may increase postoperative complications and prolong hospitalization, leading to poor treatment outcomes and increased mortality [8, 9]. Our previous studies have also demonstrated that malnutrition is associated not only with increased postoperative complications but also with poor long-term outcomes [10, 11]. In recent years, a comprehensive malnutrition index that integrates height, weight, and serum albumin levels, geriatric nutritional risk index (GNRI), has been reported to be associated with the prognosis of multiple GI malignancies, including colorectal cancer (CRC) [12], gastric cancer (GC) [13], and esophageal squamous cell carcinoma (ESCC) [14]. A previous meta-analysis proved that the GNRI could be used as a predictor of mortality [15]. Two meta-analyses have demonstrated the prognostic role of the GNRI in cancer patients, but both have certain limitations. Lv et al. [16] focused on the relationship between the GNRI and long-term prognosis of cancer patients but did not report the relationship between the GNRI and postoperative complications. Lidoriki et al. [17] comprehensively analyzed the role of the GNRI in the short-term and long-term outcomes of cancer patients but did not independently report the association between GNRI and GI malignancy. In addition, a number of other studies on the prognostic role of the GNRI in GI malignancy have been issued in the past year. Considering that there is no systematic study to determine the relationship between the GNRI and GI malignancy, the aim of this study was to systematically evaluate the value of the GNRI in postoperative complications and long-term outcomes in GI malignancy based on existing evidence.

## Methods

### Search strategy

Our meta-analysis was carried out in line with the Preferred Reporting Items for Systematic Reviews and Meta-Analyses (PRISMA) statement. We systematically searched PubMed, Web of Science, Cochrane Library, and Embase databases for literature on the value of the GNRI in assessing postoperative complications and long-term outcomes in GI malignancy. The search included literature published as of August 2020, and the literature's language was confined to English. The search terms and keywords used were as follows: “geriatric nutritional risk index”, “GNRI” and “neoplasms”, “carcinoma”, “cancer”, “tumor”. Furthermore, the reference lists of the searched literature were also reviewed to identify more potential studies.

### Inclusion and exclusion criteria

The inclusion criteria were as follows: (1) studies reported the relationship between the GNRI and postoperative complications or long-term prognosis of patients with GI malignancy and (2) the primary outcomes included postoperative complications, overall survival (OS). The secondary outcomes included relapse-free survival (RFS), disease-free survival (DFS), and cancer-specific survival (CSS). (3) Hazard ratio (HR) and 95% confidence interval (CI) were available or could be calculated based on the data provided by survival curves. (4) According to the GNRI, patients in the study were divided into two groups. The exclusion criteria were as follows: (1) reviews, case reports, conference abstracts, letters, and meta-analysis. (2) Insufficient detailed data for statistical analysis. (3) GNRI in non-GI malignancy. (4) Duplicate publication. When there were studies with the same center and the same period, the study with the most detailed data was selected.

### Data extraction and quality assessment

The variables were collected from each enrolled study: General information included the first author's last name, year of publication, country, cancer site, study design, sample, age, sex ratio, cutoff value, treatment, and analysis methods. Outcome variables included primary outcomes (complications and OS) and secondary outcomes (PFS, DFS, and CSS). If only the survival curve was provided, Engauge Digitizer v.4.1 software was used to extract the corresponding HR and 95% CI. Two independent investigators utilized the Newcastle–Ottawa Scale (NOS) to assess the quality of the eligible studies (18). If there were a difference, the disagreement was resolved through discussion with a third investigator until consensus was reached. In this study, a NOS score greater than six was considered high methodological quality.

### Statistical analysis

The extracted data were aggregated for analysis. Combined OR > 1 indicated an increased risk of postoperative complications in patients with a low GNRI. Combined HR > 1 indicated a poor long-term outcome in patients with a low GNRI. A 95% CI > 1 was considered statistically significant. Statistical heterogeneity of eligible studies was calculated by I^2^ statistics and Cochran's Q test. If I^2^ was > 50% or P_Q_ was < 0.1, the random-effects model was used for significant heterogeneity. Otherwise, the fixed-effect model was adopted. Subgroup analysis was used to assess the impact of each subgroup on the combined effect. Sensitivity analysis was used to identify the stability of results. Potential publication bias was judged by Begg’s test and Egger’s test. If there was a potential bias, the trim-and-fill method was used to reassess. A P < 0.05 was considered significant. All statistical analyses were performed through Stata (version 12.0; Stata Corp).

## Results

### Literature search

**Fig. 1:**
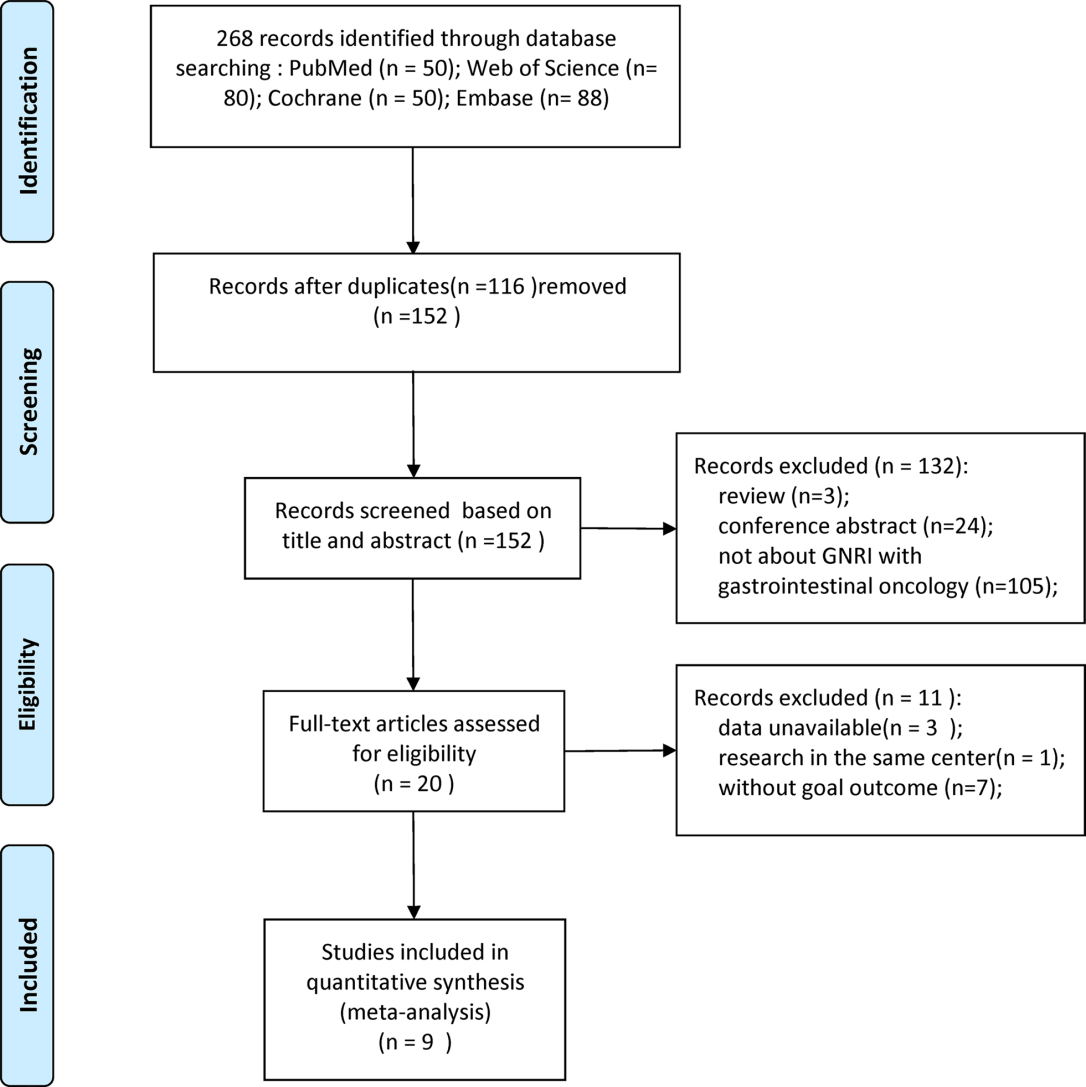
Flowchart of included studies

Figure 1 shows the literature screening process of this study. A total of 268 studies were retrieved from the databases according to the search strategy. There were 116 duplicate studies that were removed, leaving 152 studies for further screening. After carefully reading the titles and abstracts of the studies, three reviews, 24 conference abstracts, and 105 studies not about GNRI with GI malignancy were deleted. Then, the full texts of the 20 included studies were evaluated. Two of these studies were from the same center and the same period; therefore, the one with incomplete data was excluded. Three studies were unable to extract HR due to the GNRI group ≥ 3. Seven of the studies lacked relevant outcomes. Therefore, our meta-analysis included nine studies involving 2,153 patients [10, 12, 14, 19–24].

### Study characteristics

The baseline information of the nine eligible studies is listed in Table 1. All the studies were single center retrospective studies, published between 2018 and 2020, with sample sizes ranging from 80 to 348. Seven studies were from Japan, and two studies were from China. Two studies reported GC [19, 22], three studies reported ESCC [14, 20, 21], two studies reported CRC [10, 12], one study reported colorectal liver metastasis (CRLM) [24], and one study reported pancreatic ductal adenocarcinoma (PDAC). In addition, four studies reported postoperative complications, eight studies reported OS, two studies reported RFS, and two studies reported DFS and CSS, respectively. The NOS score for these nine studies ranged from 7–8.

**Table 1:** The characteristics of included studies

Study/Year	Country	Cancer site	Study design	Sample	Age(years)	Gender ratio	Cutoff value	Treatment	Outcome	Analysis	NOS
Kushiyama et al. [19]	Japan	GC	R	348	Mean 79.6 ± 3.8	230/118	92	With-surgery	Complications	M	8
Migita et al. [20]	Japan	ESCC	R	137	—	116/21	98	With-surgery	OS, RFS	U and M	7
Yamana et al. [21]	Japan	ESCC	R	216	—	—	92	With-surgery	OS	M	8
Kubo et al. [14]	Japan	ESCC	R	244	Mean 63.4	196/44	92	With-surgery	OS, CSS	M	8
Hirahara et al. [22]	Japan	GC	R	303	≥ 65	209/94	85.7	With-surgery	Complications, OS	M	8
Hu et al. [23]	China	PDAC	R	282	Mean 58.7 ± 13.5	117/165	98	With-surgery	OS	M	8
Iguchi et al. [24]	Japan	CRLM	R	80	Mean 63.6(30–86)	44/36	98	Mix	OS, RFS	M	8
Sasaki et al. [25]	Japan	CRC	R	313	Median 73(65–94)	201/112	98	With-surgery	Complications, OS	M	8
Tang et al. [10]	China	CRC	R	230	Mean 70.6 ± 5.4	154/76	98	With-surgery	Complications, OS, DFS	M	8

### GNRI and overall survival

**Fig. 2:**
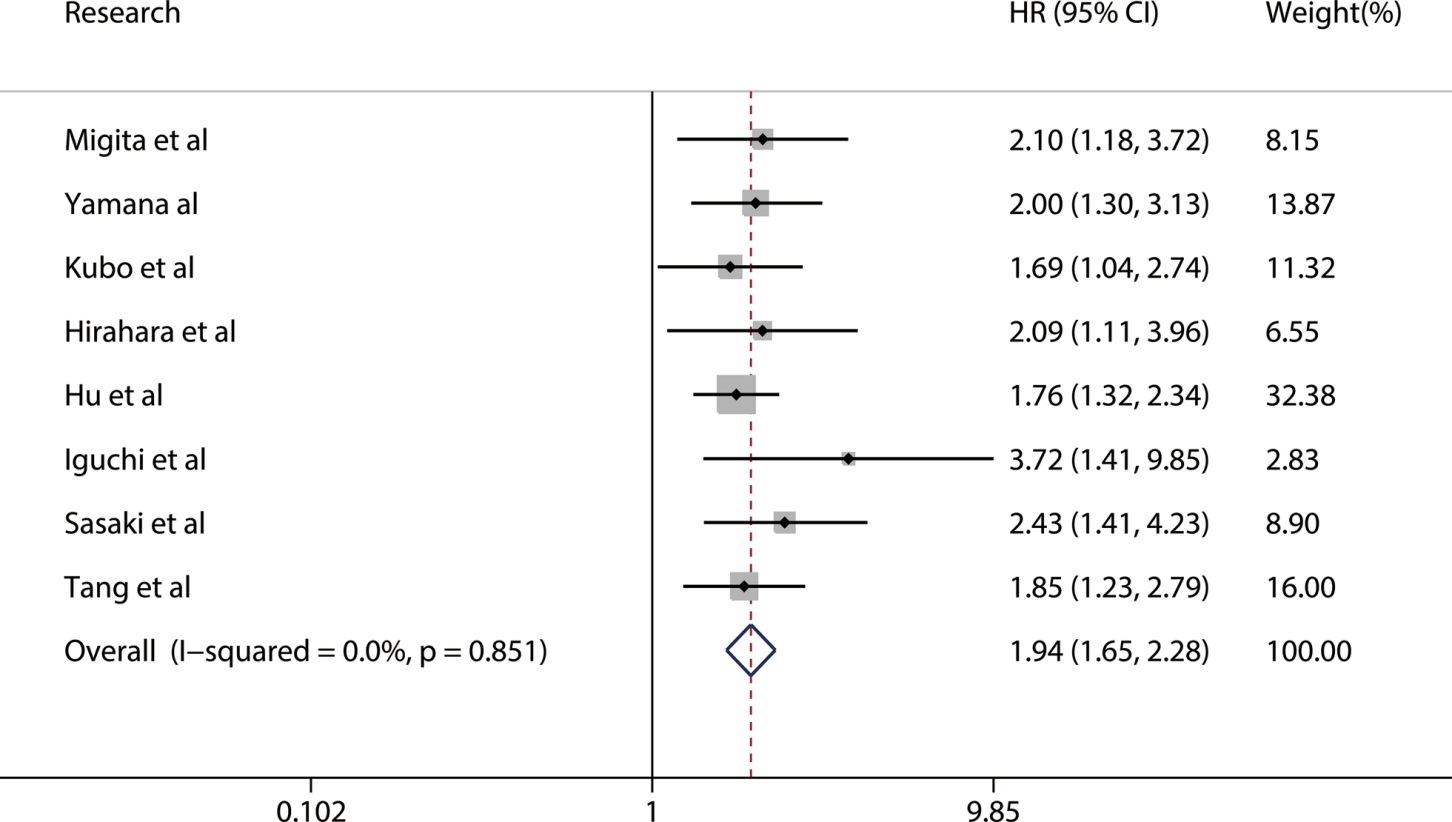
Forest plot for the association between GNRI and overall survival

**Fig. 3:**
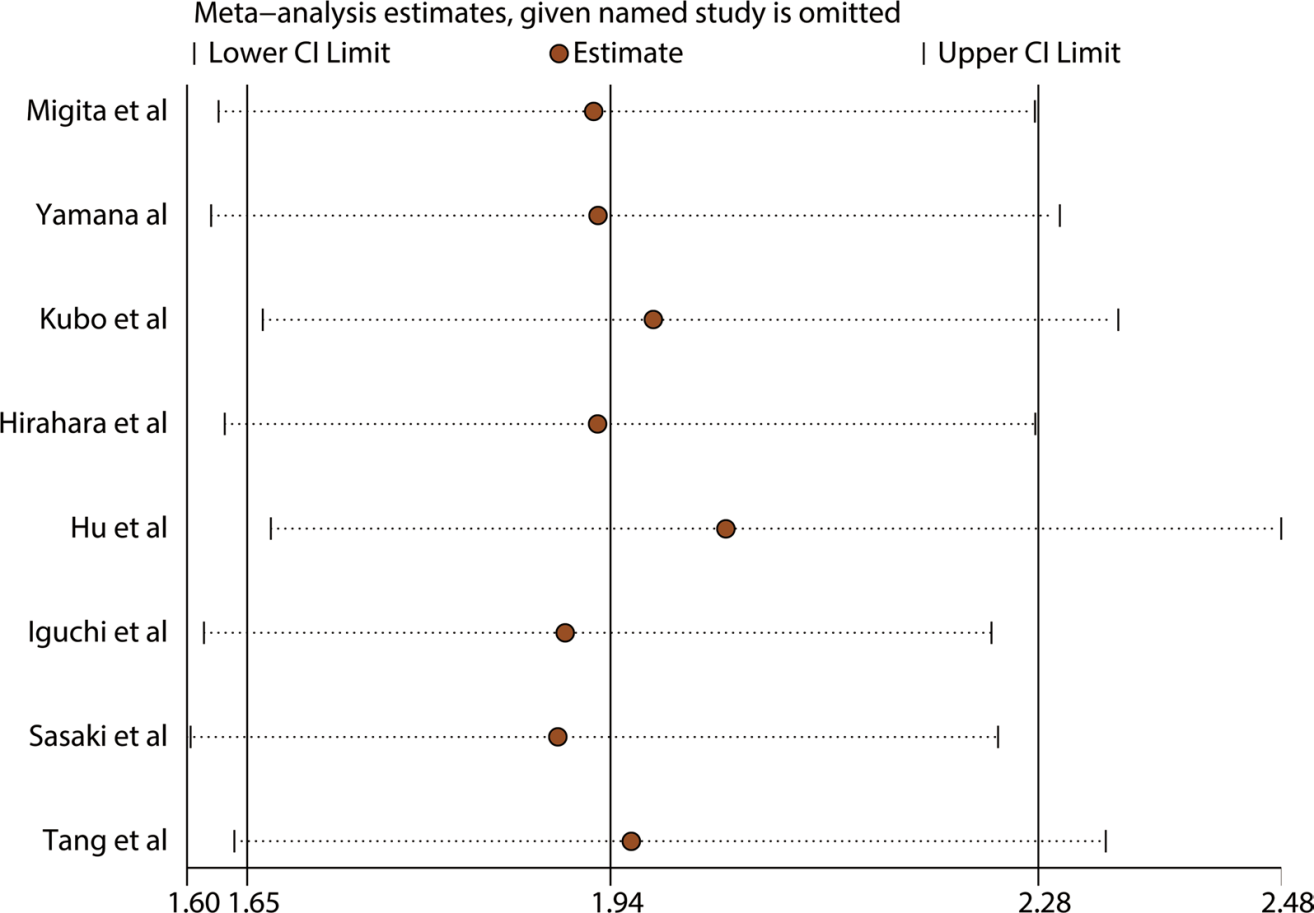
Sensitivity analysis for the association between GNRI and overall survival

**Fig. 4:**
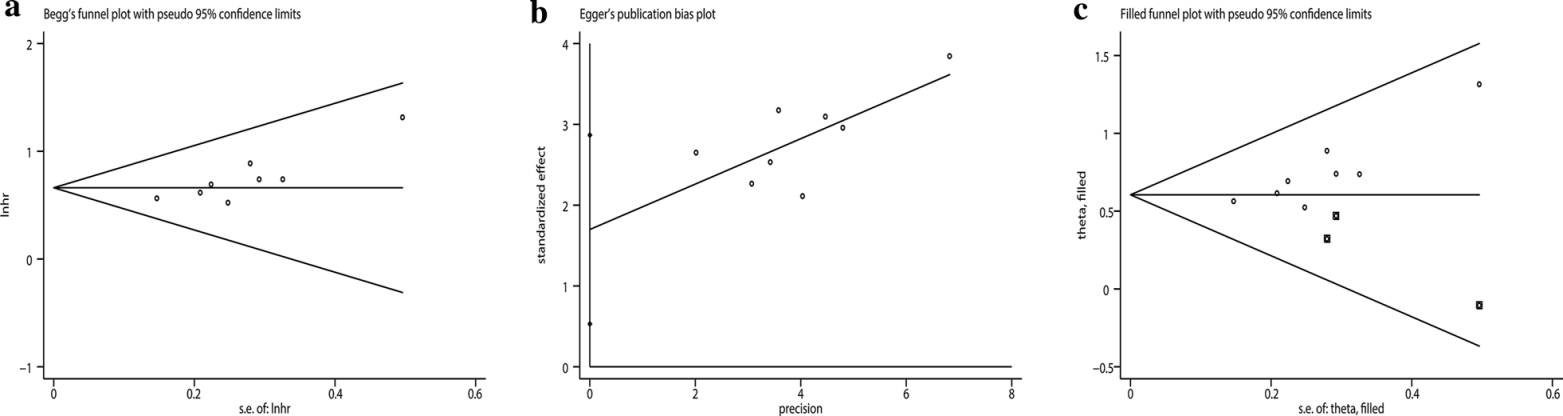
Plots for publication bias test in meta-analysis for overall survival. **a** Begg’s funnel plot; **b** Egger’s publication bias plot; **c** The trim-and-fill methods;

A total of eight studies, with 1,805 patients, reported a relationship between the GNRI and OS. The combined forest plot showed that a low GNRI was associated with poor OS in patients with GI malignancy (HR = 1.94, 95% CI 1.65–2.28, p < 0.001) (Fig. 2). Since there was no significant heterogeneity between the studies (I^2^ = 0.0%, P_Q_ = 0.851), a fixed-effects model was used. We also conducted a subgroup meta-analysis by correcting for the influence of publishing time, country, sample, cutoff value, cancer site, and primary therapy. The results indicated that the GNRI was an independent prognostic factor affecting the OS of patients in all subgroups. The results are presented in Table 2. In addition, we performed a sensitivity analysis by removing each enrolled study each time. The results showed that ignoring any of the enrolled studies did not significantly change the effect of the GNRI on the combined meta-analysis for OS. In other words, our results were robust and reliable (Fig. 3). In the meta-analysis for OS, the Begg’s test (p = 0.035) and Egger’s test (p = 0.012) determined significant publication bias (Fig. 4a, b). We further used the trim-and-fill method to correct for potential publication bias. The results revealed that three imputed studies were added to form a symmetric funnel plot (Fig. 4c), and the corrected HR was still significant (HR = 1.831, 95% CI 1.577–2.126, p < 0.001), indicating that our results were reliable.

**Table 2:** Stratification analysis of the meta-analysis for overall survival in patients with gastrointestinal malignancy

Subgroup	No. study	No. patients	Pooled HR (95% CI)	p	p for subgroup difference	Heterogeneity	
						I^2^(%)	P_Q_
Altogether	8	1805	1.94(1.65–2.28)	< 0.001		0.0	0.851
Publishing time					0.753		
< 2019	2	353	2.04(1.44–2.88)	< 0.001		0.0	0.898
≥ 2019	6	1452	1.91(1.59–2.30)	< 0.001		0.0	0.665
Country					0.351		
China	2	512	1.79(1.41–2.26)	< 0.001		0.0	0.836
Japan	6	1293	2.09(1.66–2.62)	< 0.001		0.0	0.787
Sample					0.175		
< 300	5	1295	1.84(1.53–2.20)	< 0.001		0.0	0.967
≥ 300	3	510	2.46(1.68–3.61)	< 0.001		0.0	0.623
Cutoff value					0.873		
< 98	3	763	1.90(1.42–2.54)	< 0.001		0.0	0.831
≥ 98	5	1042	1.96(1.60–2.38)	< 0.001		0.0	0.567
Cancer site					0.673		
ESCC	3	597	1.91(1.44–2.53)	< 0.001		0.0	0.821
GC	1	303	2.09(1.11–3.96)	0.023		—	—
PDAC	1	282	1.76(1.32–2.34)	< 0.001		—	—
CRLM	1	80	3.72(1.41–9.85)	0.008		—	—
CRC	2	543	2.04(1.47–2.83)	< 0.001		0.0	0.437
Primary therapy					0.181		
With-surgery	7	1725	1.90(1.61–2.24)	< 0.001		0.0	0.956
Mixed	1	80	3.72(1.41–9.85)

### GNRI and postoperative complication

**Fig. 5:**
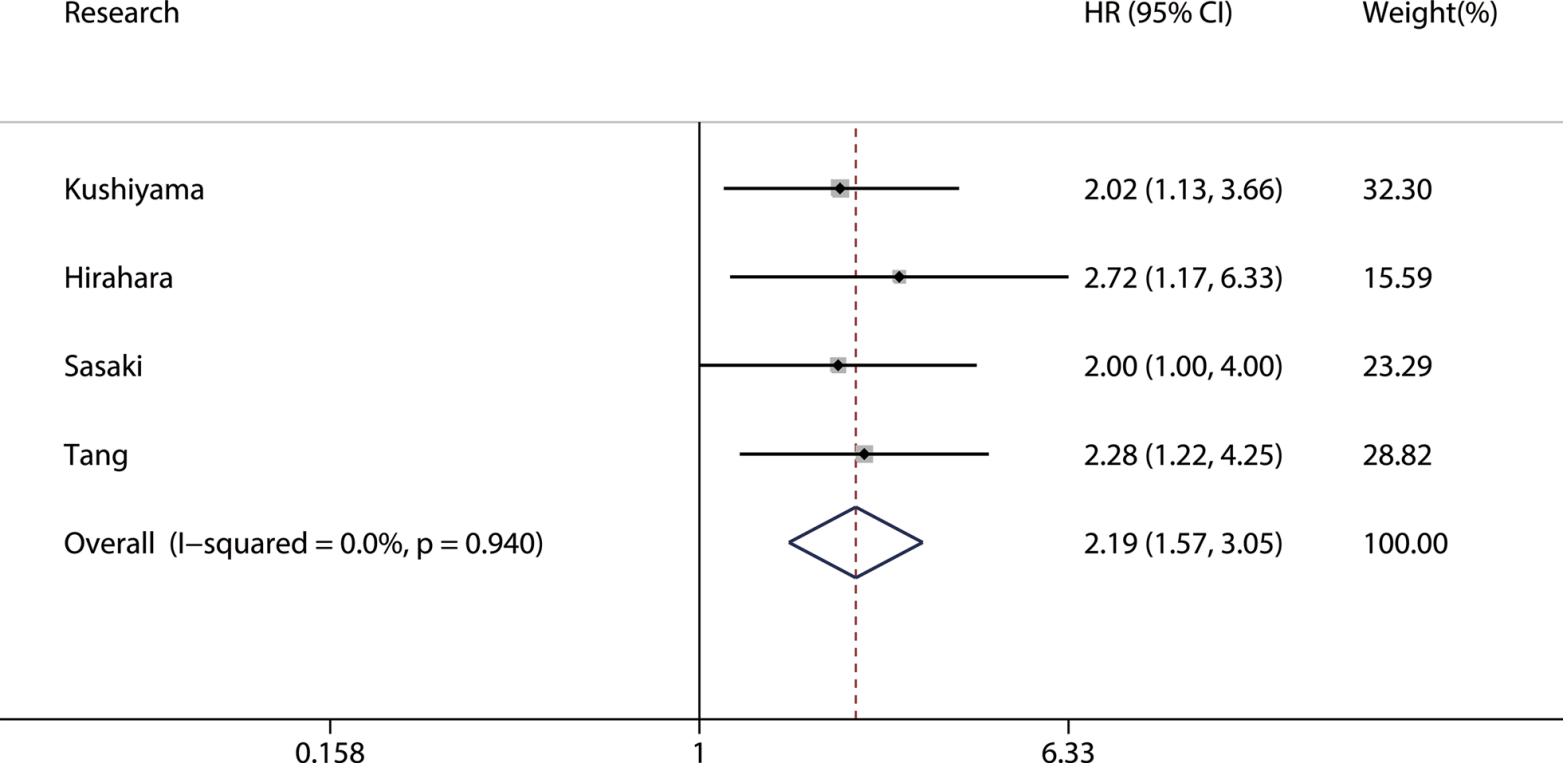
Forest plot for the association between GNRI and complications

**Fig. 6:**
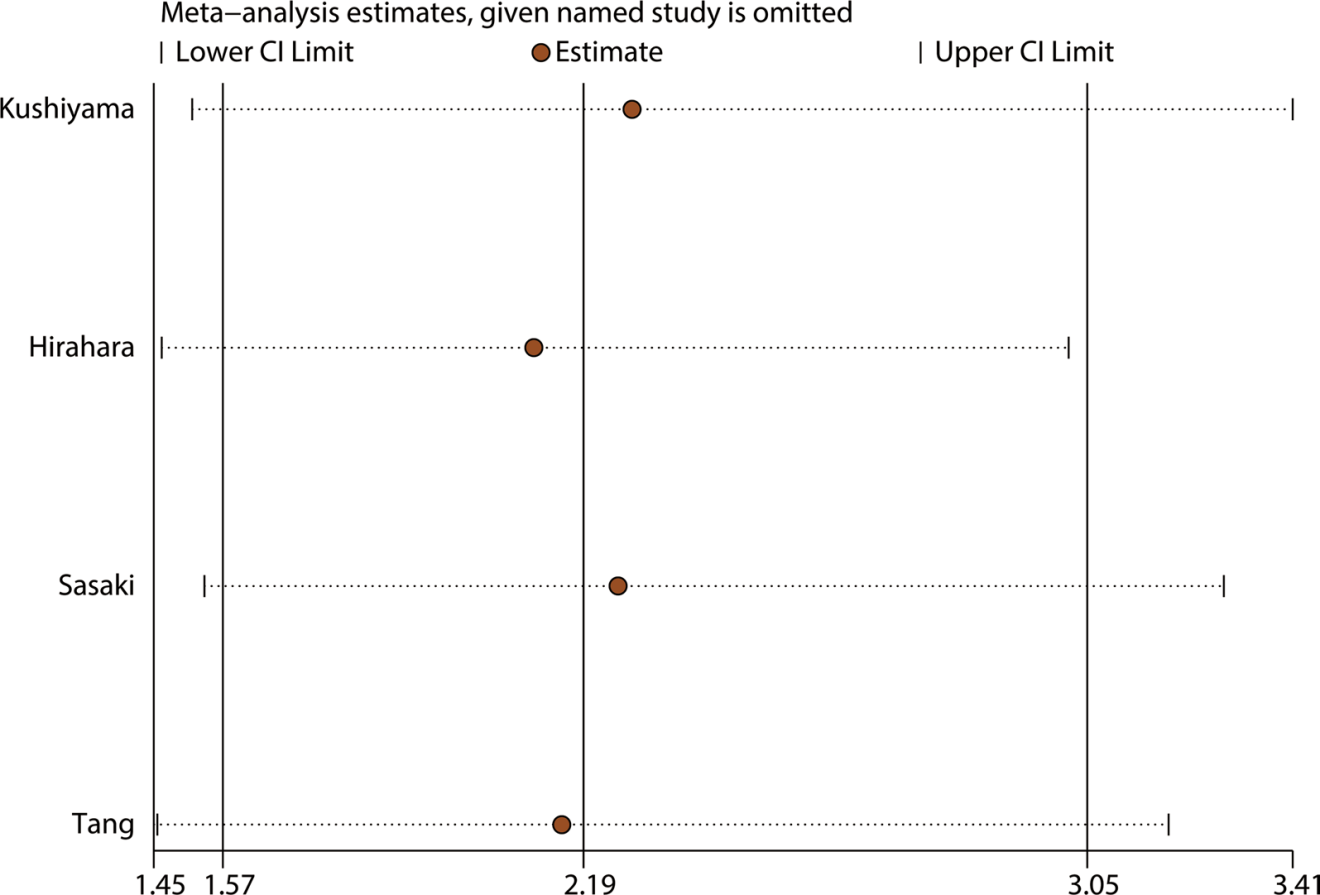
Sensitivity analysis for the association between GNRI and complications

**Fig. 7:**
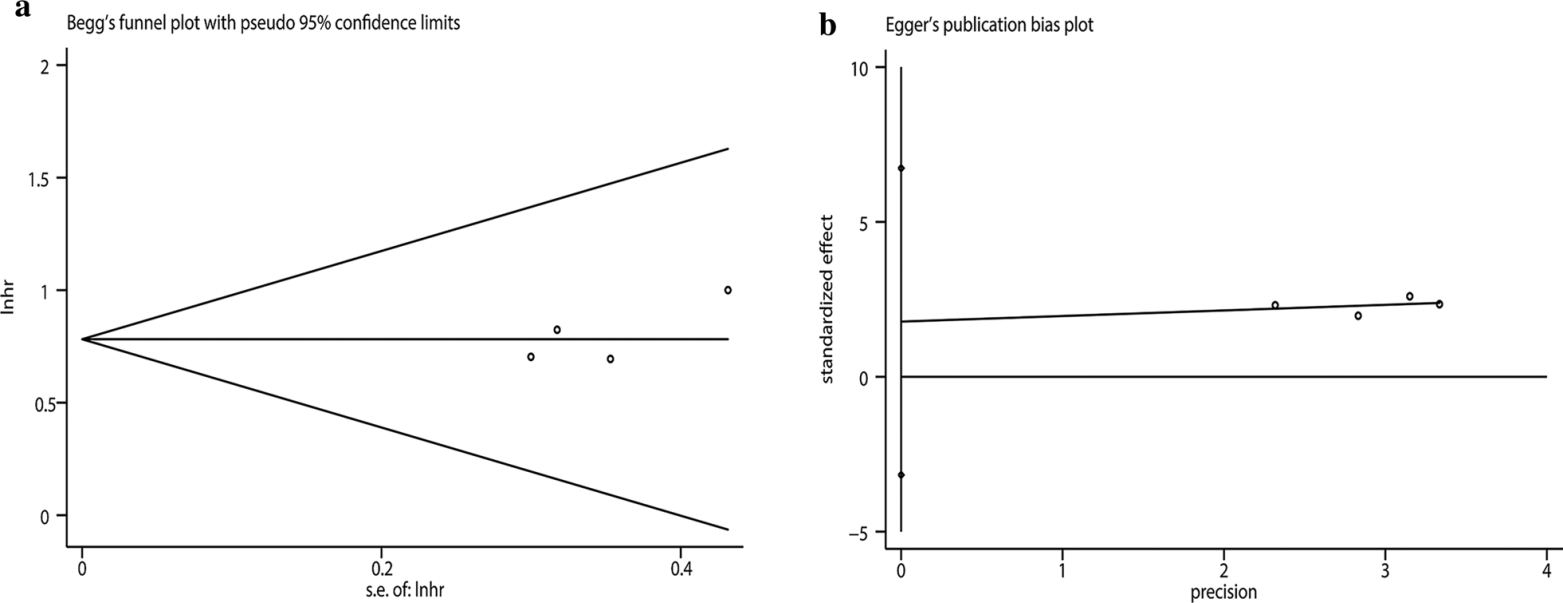
Plots for publication bias test in meta-analysis for complications. **a** Begg’s funnel plot; **b** Egger’s publication bias plot

A total of four studies involving 1,194 patients reported a relationship between the GNRI and postoperative complications in patients with GI malignancy. As shown in Fig. 5, the combined results showed that patients with a low GNRI had a higher risk of complications than those with a high GNRI (OR = 2.19, 95% CI 1.57–3.05, p < 0.001). Because there was no heterogeneity, a fixed model (I^2^ = 0.0%, P_Q_ = 0.940) was used. We performed a further subgroup analysis based on publishing time, country, sample, cutoff value, and cancer site subgroups. The results revealed that a low GNRI was an independent risk factor affecting postoperative complications in all subgroups (Table 3). We also performed a sensitivity analysis by deleting one study at a time and recalculating the combined effect. The results showed that the combined effect was not significantly changed with the deletion of any study, indicating that the results of the meta-analysis for complications were reliable (Fig. 6). In addition, Begg’s test (p = 0.308) and Egger’s test (p = 0.262) both suggested that there was no potential publication bias in the meta-analysis for complications (Fig. 7).

**Table 3:** Stratification analysis of the meta-analysis for complications in patients with gastrointestinal malignancy

Subgroup	No. study	No. patients	Pooled HR (95% CI)	p	p for subgroup difference	Heterogeneity	
						I^2^(%)	P_Q_
Altogether	4	6035	2.19(1.57–3.05)	< 0.001		0.0	0.94
Publishing time					0.921		
< 2019	2	2171	2.22(1.37–3.60)	0.001		0.0	0.573
≥ 2019	2	3864	2.15(1.35–3.42)	0.001		0.0	0.783
Country					0.875		
China	1	5788	2.28(1.22–4.25)	0.009		—	—
Japan	3	83	2.15(1.45–3.19)	< 0.001		0.0	0.828
Sample					0.875		
< 300	1	1295	2.15(1.45–3.19)	0.009		—	—
≥ 300	3	4740	1.81(1.58–2.07)	< 0.001		0.0	0.828
Cutoff value					0.921		
< 98	2	3274	2.22(1.37–3.60)	0.001		0.0	0.573
≥ 98	2	2761	2.15(1.35–3.42)	0.001		0.0	0.783
Cancer site					0.921		
GC	2	888	2.22(1.37–3.60)	0.001		0.0	0.573
CRC	2	6035	2.15(1.35–3.42)	0.001		0.0	0.783

### GNRI and RFS/DFS/CSS

**Fig. 8:**
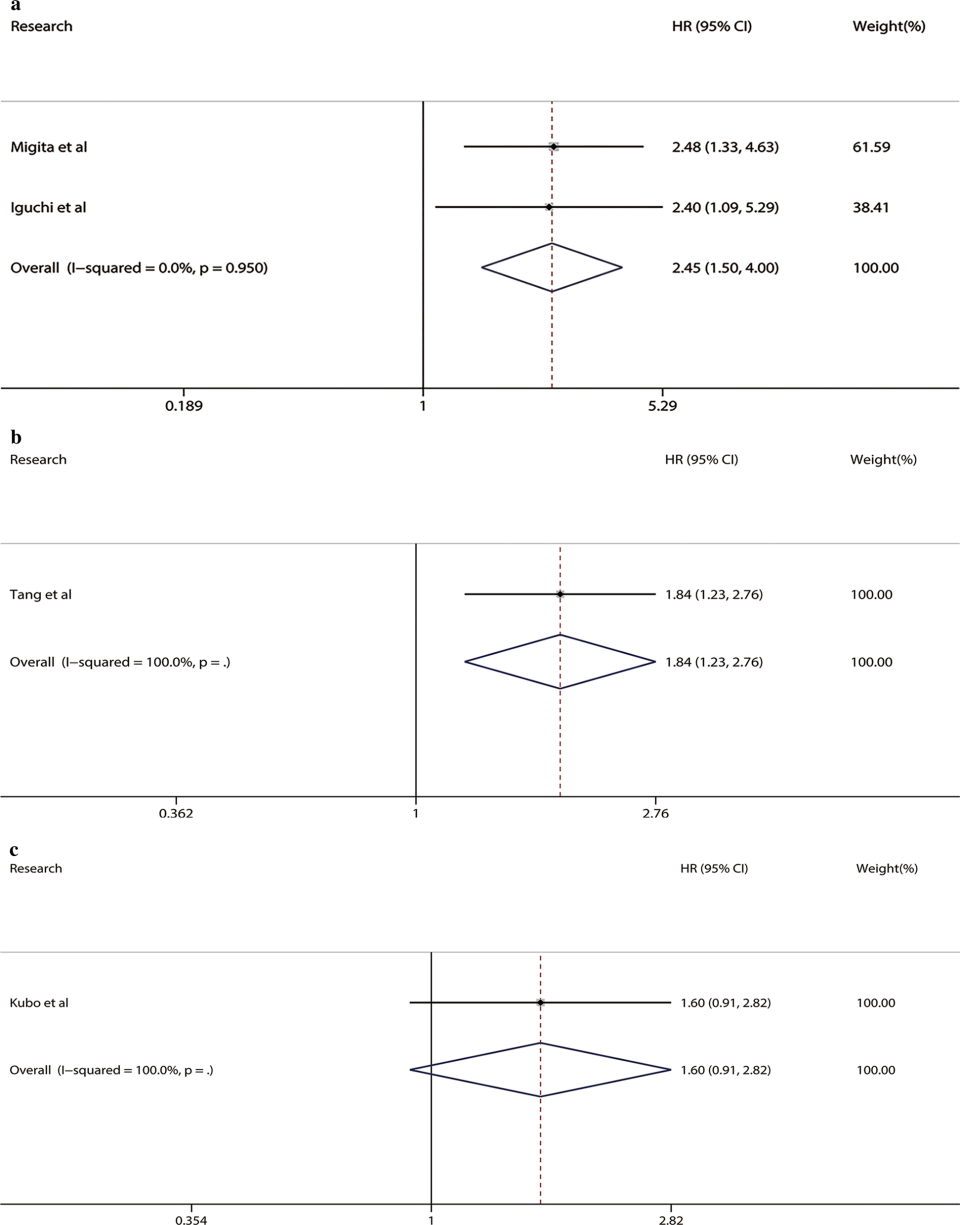
Forest plot for the association between GNRI and relapse-free survival (**a**)/disease-free survival (**b**)/cancer-specific survival (**c**)

We also explored the effects of the GNRI on RFS/DFS/CSS in patients with GI malignancy. Two studies involving 217 patients reported a relationship between the GNRI and RFS. The combined results showed that patients with a low GNRI had shorter RFS (HR = 2.45, 95% CI 1.50–4.00, p < 0.001) than patients with a high GNRI, and the heterogeneity between studies was not significant (I^2^ = 0.0%, P_Q_ = 0.950), as shown in Fig. 8a. Similarly, a study involving 230 patients confirmed that a low GNRI was an independent risk factor for DFS in patients with GI malignancy (HR = 1.84, 95% CI 1.23–2.76, p = 0.003) (Fig. 8b). However, a study involving 244 patients showed that the GNRI was not an independent predictor of CSS for patients with GI malignancy (HR = 1.60, 95% CI 0.91–2.82, p = 0.101) (Fig. 8c).

## Discussion

In 2005, Bouillanne et al. [25] established the GNRI based on commonly used clinical parameters as a nutrition-related prognostic assessment tool for inpatients. Since then, the GNRI has been widely used to evaluate the prognosis of elderly patients with chronic liver failure, chronic obstructive pulmonary disease, cardiovascular disease, and other chronic diseases. Over the past 5 years with the development of the tumor nutrition theory, the GNRI, as a simple, inexpensive, and readily available biomarker, has been gradually applied to evaluate the prognosis of cancer patients. It was not until 2018 that many studies emerged to explore the relationship between the GNRI and clinical outcome of GI malignancy. As shown in Table 1, most studies included in our meta-analysis were published between 2018 and 2020. In other words, the GNRI is still a very novel index in the field of GI malignancy. Hirahara et al. [22] suggested that perioperative nutritional support based on a preoperative GNRI assessment could reduce the incidence of postoperative complications in patients with gastric cancer, thus improving their long-term prognosis. A study of 240 patients reported that the GNRI is a more independent prognostic factor for ESCC patients than body mass index (BMI) and albumin alone [14]. Sasaki et al. [12] also proposed that the GNRI is an independent prognostic factor for the short-term and long-term prognosis of CRC independent of TNM stage. In our previous studies, the authors believed that the GNRI could help identify whether patients were truly malnourished and that the GNRI was more suitable than other nutrition-related indicators (prognostic nutritional index, BMI, and albumin) to assess the prognostic value in elderly CRC patients. In addition, studies have reported that the GNRI is a simple and useful predictor of sarcopenia, which is often regarded as an important adverse prognostic factor in GI malignancy. The GNRI may be more clinically useful than sarcopenia because it is easier to measure and obtain [26].

It is well-known that malnutrition may increase the incidence of postoperative complications in cancers and adversely affect long-term survival. Timely nutritional support can reduce postoperative morbidity and hospitalization rates [27, 28]. It has been reported that decreased BMI is associated with an increased risk of disease progression and death in patients with GI malignancy [29]. The GNRI integrates weight-related information, which may more objectively reflect the weight change in tumor patients due to tumor consumption. Hypoalbuminemia has been proven to be a poor prognostic factor for a variety of malignancies [30]. It may be associated with decreased albumin, which inhibits the body's ability to cope with stresses such as malignancy and surgery. This would lead to an inadequate anti-tumor immune response and reduced wound healing ability, thereby increasing the risk of postoperative complications and a poor prognosis [31]. The GNRI, combined with these factors, is a useful comprehensive indicator of nutritional and immune status.

In this study, we included nine studies with a total of 2153 patients with GI malignancy. We found that the GNRI is an independent factor influencing the long-term outcome of patients with GI malignancy. The consistent results of sensitivity and subgroup analysis showed that our results were reliable and robust. Although publication bias was detected, we utilized the trim-and-fill method to correct the bias. The results showed that it did not change the significant correlation between a low GNRI and poor OS, indicating that our conclusion was robust. We have further explored the relationship between GNRI and postoperative complications. The combined results showed that the GNRI was an independent influencing factor for postoperative complications in patients with GI malignancy. At the same time, a stratified meta-analysis showed that despite differences in publication year, country, sample size, cutoff value, and cancer site among different groups, a low GNRI was still significantly associated with postoperative complications. Moreover, the sensitivity analysis suggested that the results of mate-analysis for complications were reliable, and there was no publication bias in the mate-analysis for complications. In addition, we also found a significant association between a low GNRI and unfavorable RFS/DFS in GI malignancy, while no statistical association was found between a low GNRI and adverse CSS. Based on these results, the GNRI may be considered an effective and practical clinical indicator for predicting the short-term and long-term outcomes of patients with GI malignancy.

To our knowledge, our study is the first meta-analysis to comprehensively explore the value of the GNRI in postoperative complications and long-term outcomes of patients with GI malignancy. We synthesized the existing evidence to prove that the GNRI has a good value in predicting the prognosis of patients with GI malignancy. Our study provides evidence-based support for the clinical application of the GNRI in the prognosis evaluation of patients with GI malignancy. In addition, the critical value of the GNRI in most of the included studies was 98, which may provide some reference value for determining the critical value of the GNRI in clinical applications. We believe that our meta-analysis can provide some inspiration for further research on the relationship between the GNRI and GI malignancy. However, this study still has some limitations. First, all the recruited studies were single center retrospective studies, and the total number of samples and studies was relatively small. More prospective randomized controlled trials are required to investigate the value of the GNRI in GI malignancy. Second, due to the limited number of enrolled studies, the value of the GNRI in RFS/DFS/CSS in GI malignancy still needs to be explored. Finally, this study only included research on all complications. The relationship between the GNRI and specific complications, such as respiratory complications, infectious complications, and anastomotic leakage, still needs to be explored.

## Conclusion

Based on existing evidence, this meta-analysis confirmed that the GNRI was a valuable predictor of complications and long-term outcomes in patients with GI malignancy. Of course, large, multicenter prospective queues are still needed to validate our findings.

## Data Availability

Please contact author for data requests.

## Abbreviations

GNRI: Geriatric nutritional risk index
95% CI: 95% Confidence interval
NOS: Newcastle–Ottawa Scale
OS: Overall survival
CSS: Cancer-specific survival
DFS: Disease-free survival
RFS: Recurrence-free survival
